# Association of KCNJ11 rs5219 gene polymorphism with type 2 diabetes mellitus in a population of Syria: a case-control study

**DOI:** 10.1186/s12881-019-0846-3

**Published:** 2019-06-13

**Authors:** Osama Makhzoom, Younes Kabalan, Faizeh AL-Quobaili

**Affiliations:** 1 Clinical Biochemistry Department, Faculty of Pharmacy, Damascus University, Damascus, Syria; 2 Endocrinology Department, Faculty of Medicine, Damascus University, Damascus, Syria

**Keywords:** KCNJ11, Polymorphism, rs5219, RFLP, Potassium channels, Beta cells, Type 2 diabetes, Syria

## Abstract

**Background:**

Type 2 diabetes mellitus is believed to be a polygenic disorder that develops as a result of a complex interaction between multiple genes and environmental factors. KCNJ11 gene encodes a Kir6.2 protein which forms the inner section of the potassium channels in pancreatic beta cells. Several studies found that KCNJ11 polymorphism increases T2DM risk. Our study aimed to investigate the association between rs5219 polymorphism of the KCNJ11 gene and T2DM in Syrian patients.

**Methods:**

This case-control study involved 75 T2DM patients and 63 healthy controls. The KCNJ11 rs5219 polymorphism was genotyped by Restriction Fragment Length Polymorphism (RFLP).

**Results:**

The frequency of the risk allele K was similar between the two groups (38.7% vs. 38.1%, *P* = 0.132). The frequency of the KK genotype was higher among the patients’ group (16% vs. 4.8%), and the frequency of the EK genotype was higher among the control group (45.3% vs. 66.6%); however, the differences were statistically insignificant. The KK genotype was significantly associated with T2DM in the recessive model with an OR of 3.81 (95% CI 1.024–14.17, *P* = 0.035).

**Conclusions:**

This study showed that rs5219 polymorphism of the KCNJ11 gene is an important risk factor for type 2 diabetes mellitus in a sample of the Syrian population.

## Background

The global rate of diabetes has increased significantly over the past two decades, reaching 451 million in 2017, and it is expected to reach more than 693 million in 2045 [1]. The Center for Disease Control and Prevention (CDC) in the United States of America had published that about 30.3 million people, equivalent to 9.4% of the US population, were diabetic as of 2015 [2]. In Syria, the proportion of people with diabetes was 11.9% in 2016 based on a World Health Organization report [3].

T2DM is believed to be a polygenic disorder that develops as a result of a complex interaction between multiple genes and environmental factors. This genetic component is more likely to be due to single-nucleotide polymorphisms (SNPs) [4]. Studies have identified several genes that may be associated with T2DM; among these, the Potassium Voltage-Gated Channel Subfamily J Member 11 gene, which has received significant attention as an important candidate gene for T2DM risk, due to its function in the regulation of glucose-induced insulin secretion [5]. The KCNJ11 gene is located at 11p15.1 and contains one exon that encodes Kir6.2 protein which forms the inner section of the adenosine triphosphate sensitive potassium ion channel (KATP) in pancreatic beta cells, and plays a crucial role in insulin secretion. Several SNPs of the KCNJ11 gene have been detected, among them, rs5219, which has been receiving more attention for its association with diabetes. KCNJ11 rs5219 polymorphism is caused by a switch of guanine to adenine at codon 23, resulting in a glutamic acid to lysine amino acid substitution and thereby a critical inhibition of glucose-induced insulin secretion. This alteration reduces potassium channels’ sensitivity to ATP molecules, resulting in over-activity of the channel and subsequent inhibiting insulin secretion [6–8].

Several studies have observed an association between KCNJ11 rs5219 polymorphism and T2DM risk. However, there are inconsistent results in previous studies in Asian populations [7–9], and there is no study has been carried out in a population of Syria, so we found it is important to investigate the association between KCNJ11 rs5219 polymorphism and T2DM in the Syrian population.

## Methods

### Study population

The study is a case-control study. It included 138 participants of Syrian Arab ethnicity, aged above 40 years. The case group included 75 T2DM patients (38 men and 37 women; median age 47.4 years), who were diagnosed according to the American Diabetes Association criteria (a fasting plasma glucose more than 126 mg/dl, or a plasma glucose more than 200 mg/dl after 2-h of oral glucose (1.75 g/kg) or HbA1c ≥ 6.5%) [10]. The control group included 63 individuals (32 men and 31 women; median age 47.9 years), who were apparently healthy with a fasting plasma glucose less than 100 mg/dl, HbA1c < 5.7%, and a negative family history of T2DM. The exclusion criteria included the patients with type I diabetes, pancreatitis, chronic gastrointestinal diseases associated with poor absorption, cancers, liver failure, or other clinical conditions likely to cause hyperglycemia such as; infections, thyroid disease, surgeries, medications which affect glucose levels. This case-control study was approved by the ethics committee of the University of Damascus, and written informed consent was obtained by all participants. The study was carried out at the laboratories of the biotechnology research center at Al-Baath University in the period from November 2016 to May 2018.

### Sampling

Samples of 5 ml venous blood (2 ml on a dry tube and 3 ml divided into two EDTA tubes) were taken after fasting for about 10 h. The dry tube was centrifuged at 1680 Xg for 10 min, and the glucose level was directly measured. The first EDTA tube was used to measure the hemoglobin A1c, which was stored at 4 °C for 2–7 days until the time of the assay, and the second was used to isolate the DNA and study the genetic variation.

### Biochemical assays

Glucose level was assayed by an enzyme-based method (Glucose Oxidase Peroxidase) using the Biosystems kit (Spain), and HbA1c was assayed by an ion exchange resin method using the Biosystems kit (Spain).

### Molecular genotyping

DNA was isolated from the whole blood using the GF-1 Blood DNA extraction kit (Vivantis, Malaysia). The concentration and purity of the isolated DNA were measured using the Biospecnano device (Shimadzue, Japan). The isolated DNA was stored at − 20 °C for polymerase chain reaction (PCR). KCNJ11-rs5219 polymorphism was genotyped by restriction fragment length polymorphism (RFLP), and PCR reaction was performed using TECHNE TC512, Gradient Thermal Cycler (Bibby Scientific, UK). A specific area of ​​the KCNJ11 gene was amplified (210 bp) using PCR Master Mix (2X) (Genedirex, Malaysia), and the following primers:

Forward: (5`-GACTCTGCAGTGAGGCCCTA-3’)

Reverse: (5`-ACGTTGCAGTTGCCTTTCTT-3’)

The PCR was carried out under the following conditions: Initial denaturation at 95 °C for 5 min, followed by 35 cycles of (a) 95 °C for 30 s (denaturation), (b) 60 °C for 30 s (annealing), (c) 72 °C for 30 s (elongation) and final elongation at 72 °C for 9 min.

The amplified DNA fragments (210 bp) were digested using the BanII enzyme (NEB, UK) at 37 °C for 2 h. The reaction volume (10 μl) contained 5 μl of PCR product, 1 μl 10X NEB buffer, 0.5 μl BanII enzyme and 3.5 μl nuclease free water. The digested products were separated by electrophoresis on a 2% agarose gel with ethidium bromide and visualized under a UV transilluminator.

### Statistical study

The statistical program (SPSS 16) was used for statistical analysis. The chi-square test was used to determine whether the genotype distributions were in Hardy-Weinberg equilibrium. The frequency of the genotypes and alleles were compared between the two groups using chi-square test. The odds ratios were calculated using a logistic regression model. *P-value* <  0.05 was considered statistically significant.

## Results

The clinical and biological characteristics of the participants are summarized in (Table 1). Significant differences were observed for body mass index (BMI), fasting plasma glucose, and hemoglobin A1c. There were no statistically significant differences for sex and age.

**Table 1:** The clinical and biological characteristics of the participants

Variables	Patients (*n* = 75)	Controls (*n* = 63)	*P-value*
Age (years)	47.4 ± 5.2	47.9 ± 7.1	0.923
Sex (male/female)	38/37	32/31	0.9
Body mass index (kg/m^2^)	26.1 ± 1.7	23.9 ± 2.4	0.021
Fasting plasma glucose (mg/dl)	192.32 ± 33.4	91.1 ± 11.2	< 0.0001
HbA1c %	8.6 ± 0.9	5.1 ± 0.2	< 0.0001

The DNA concentration of all samples ranged from 10 to 65 ng/μl with an average of 35 ng/Μ*l. index* of purity ranged from (1.7 to 1.9).

**Fig. 1:**
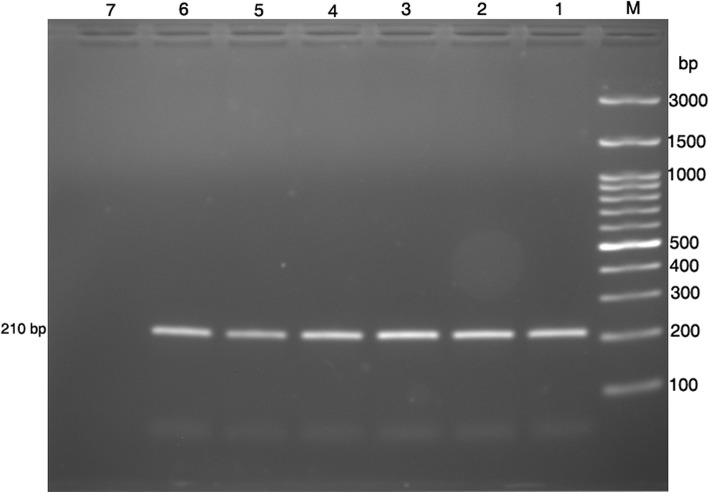
The product of the PCR amplification of KCNJ11- rs5219. Samples were electrophoresed on a 2% agarose gel. Lane M: 100 bp ladder, Lane (1,2,3,4,5,6): PCR products (210 bp), Lane 7: negative control

When a PCR reaction was applied, a band of DNA was produced with a length of 210 bp, (Fig. 1).

**Fig. 2:**
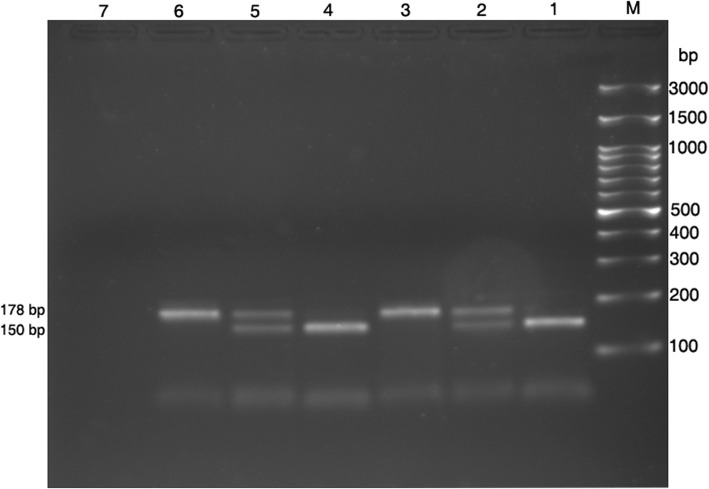
Digestion products for KCNJ11- rs5219 by BanII enzyme. Samples were electrophoresed on a 2% agarose gel. Lane M: 100 bp ladder, Lane (1,4): homozygote EE, Lane (2,5): heterozygote EK, Lane (3,6): homozygote KK

When applying the BanII enzyme on the PCR product, different patterns were obtained:

- One band of 150 bp for the wild type homozygote EE.
- One band of 178 bp for the mutant homozygote KK.
- Two bands of 178 bp and 150 bp for heterozygote EK, (Fig. 2).

The frequency of (EE-EK-KK) genotypes for the patients’ group was (38.7–45.3% - 16%), and for the control group (28.6–66.6% - 4.8%), respectively. The difference between the two groups was statistically significant, (*P-value* = 0.02).

Genotype frequencies for all participants were in Hardy-Weinberg equilibrium.

The frequency of (E – K) alleles was (61.3–38.7%) in the patients’ group, and (61.9–38.1%) in the control group, respectively. The difference was shown to be statistically insignificant, (*P*-value = 0.923).

Four models were tested to evaluate the effect of KCNJ11- rs5219 polymorphism on T2DM, and odds ratio and *P-value* were calculated for each model as follows:

- Homozygote model (KK vs. EE): OR: 2.483 (95% CI 0.615–10.02, *P* = 0.192)
- Heterozygote model (EK vs. EE): OR: 0.502 (95% CI 0.239–1.055, *P* = 0.067)
- Dominant model (EK + KK vs. EE): OR: 0.634 (95% CI 0.31–1.3, *P* = 0.213)
- Recessive model (KK vs. EE + EK): OR: 3.81 (95% CI 1.024–14.17, *P* = 0.035), (Table 2).

**Table 2:** Genetic characteristics of the T2DM patients and control groups

Variables	Patients (*n* = 75)	Controls (*n* = 63)	OR (95% CI)	*P-value*
Genotypes				
E/E	29 (38.7%)	18 (28.6%)	Reference	
E/K	34 (45.3%)	42 (66.6%)	0.502 (0.239–1.055)	0.067
K/K	12 (16%)	3 (4.8%)	2.483 (0.615–10.02)	0.192
Dominant model				
E/E	29 (38.7%)	18 (28.6%)	Reference	
E/K + K/K	46 (61.3%)	45 (71.4%)	0.634 (0.31–1.3)	0.213
Recessive model				
E/E+ E/K	63 (84%)	60 (95.2%)	Reference	
K/K	12 (16%)	3 (4.8%)	3.81 (1.024–14.17)	0.035
Alleles				
E	92 (61.3%)	78 (61.9%)	Reference	
K	58 (38.7%)	48 (38.1%)	1.024 (0.629–1.667)	0.923

## Discussion

T2DM is one of the most prevalent non-infectious diseases worldwide and is a major healthcare problem. The genetic factors behind the T2DM are believed to be multiple and complex in nature [4]. Therefore, there is a need to investigate these genetic factors which are associated with the risk of T2DM. Since the genetic studies of T2DM in the Middle East region are limited, we have studied the association between rs5219 polymorphism of the KCNJ11 gene and T2DM in a sample of the Syrian population.

We found that the difference between the three genotypes of KCNJ11-rs5219 polymorphism (EE- EK- KK) in the two groups was statistically significant, (*P* = 0.02).

The prevalence of the K allele was 38.4% in our study. This is comparable to studies in Caucasian and Asian populations [9–15]. However, the K allele frequency was lower in North African populations [16–18]. This suggests a moderate difference in the risk allele K according to ethnicity and geographic location, (Table 3).

**Table 3:** The prevalence of the K allele among different populations

Author	Year	Country	Ethnicity	Case	Control	K%
Anna L Gloyn	2001	UK	Caucasian	364	328	37%
Eva-Maria D Nielsen	2003	Denmark	Caucasian	803	862	39%
Martine Vaxillaire	2008	France	Caucasian	307	2919	39%
Yuki Sakamoto	2007	Japan	Asian	909	893	36%
Daizhan Zhou	2009	China	Asian	1912	2041	40%
Parvaneh Keshavarz	2014	Iran	Asian	400	420	36%
Khaled Lasram	2014	Tunisia	North African	250	267	22%
Isselmou Abdelhamid	2013	Mauretania	North African	135	135	19%
Houda Benrahma	2014	Morocco	North African	250	250	20%

The frequency of the KK genotype was higher among the patients’ group, whereas the frequency of the EK genotype was higher among the control group. Studies of Iranian and Russian populations found the same results [19, 20].

When assuming a variety of models to evaluate the association between KCNJ11 rs5219 polymorphism and T2DM risk, the recessive model (KK vs. EE + EK) was associated with the T2DM risk with an OR of 3.81 (95% CI 1.024–14.17, *P* = 0.035). Consequently, the KK genotype is associated with an increase in the risk of T2DM by four times.

This finding is in accord with a meta-analysis study (Wang et al., 2018) [7] which found that the recessive model is the most appropriate model for evaluating the effect of this gene variation on the risk of T2DM.

In a study of the Russian population, the KK genotype was associated with an increase in the risk of T2DM by two and a half times [20], and in studies of Mauritanian and German populations by two times [17–21], and in studies of Iranian and Chinese populations by one and a half times [19–22], and in a meta-analysis study (Wang et al., 2018) by one and a quarter times.

It is known that T2DM is a complex disorder caused by the interaction of multiple genetic and environmental factors, and the effects of the same genetic factor on T2DM development are not the same among people, due to their difference in the environmental elements. Many people in Syria suffer from generally poor nutritional habits and a lack of health awareness, in addition to the stress that most people have suffered as a result of the years of war. As these strong environmental elements may increase the impact of the genetic factor in the risk allele carriers, this may explain the high-risk value (four times) in our study in a sample of the Syrian population compared to other populations.

This is proven by Keshavarz et al [9], where the study of an Iranian population did not find an association between rs5219 polymorphism of the KCNJ11 gene and type 2 diabetes. However, when the results were limited to obese individuals, an association has been shown. This may confirm the increasing effect of the genetic factor in the presence of strong environmental elements.

The high-risk value may also be explained by the sample size (138 participants), which is small for such types of studies, as we noticed very big confidence intervals for odds ratio “OR 3.81 (95% CI 1.024-14.17)”. Therefore, the high-risk value may not reflect the reality accurately, and larger studies are needed.

In the control group, we have excluded individuals with positive family history of T2DM and this could reduce the chance of the controls to have the SNPs conferring risks to T2DM, including KCNJ11- rs5219 polymorphism, and subsequently may lead to bias in the selection of individuals. This may also explain the high-risk value in our study.

## Conclusions

This study showed an association between rs5219 polymorphism of the KCNJ11 gene and type 2 diabetes mellitus in a sample of the Syrian population. This study supports the role of KCNJ11 rs5219 polymorphism in the pathogenesis of T2DM. Larger studies should be performed to confirm this result.

## Data Availability

The datasets used and/or analyzed during the current study are available from the corresponding author on reasonable request.

## Abbreviations

CDC: Center for Disease Control and Prevention
CI: Confidence Interval.
HbA1c: Hemoglobin A1c
KCNJ11: Potassium Voltage-Gated Channel Subfamily J Member 11
OR: Odds Ratio
T2DM: Type 2 diabetes mellitus
